# Distinct rates and patterns of spread of the major HIV-1 subtypes in Central and East Africa

**DOI:** 10.1371/journal.ppat.1007976

**Published:** 2019-12-06

**Authors:** Nuno R. Faria, Nicole Vidal, José Lourenco, Jayna Raghwani, Kim C. E. Sigaloff, Andy J. Tatem, David A. M. van de Vijver, Andrea-Clemencia Pineda-Peña, Rebecca Rose, Carole L. Wallis, Steve Ahuka-Mundeke, Jean-Jacques Muyembe-Tamfum, Jérémie Muwonga, Marc A. Suchard, Tobias F. Rinke de Wit, Raph L. Hamers, Nicaise Ndembi, Guy Baele, Martine Peeters, Oliver G. Pybus, Philippe Lemey, Simon Dellicour

**Affiliations:** 1 Department of Zoology, University of Oxford, Oxford, United Kingdom; 2 TransVIHMI, Institut de Recherche pour le Développement, INSERM, and University of Montpellier, Montpellier, France; 3 Amsterdam Institute for Global Health and Development, Department of Global Health, Amsterdam University Medical Centers, University of Amsterdam, Amsterdam, The Netherlands; 4 Department of Internal Medicine, Section of Infectious Diseases, VU University Medical Center, Amsterdam University Medical Centers, University of Amsterdam, Amsterdam, The Netherlands; 5 Department of Geography and Environment, University of Southampton, Southampton, United Kingdom; 6 Flowminder Foundation, Stockholm, Sweden; 7 Viroscience Department, Erasmus Medical Center, Rotterdam, The Netherlands; 8 Global Health and Tropical Medicine—Instituto de Higiene e Medicina Tropical, Universidade Nova de Lisboa, Lisboa, Portugal; 9 Molecular Biology and Immunology Department, Fundación Instituto de Inmunología de Colombia, Basic Sciences Department, Universidad del Rosario, Bogotá, Colombia; 10 Bioinfoexperts, LLC, Thibodaux, Los Angeles, United States of America; 11 Department of Molecular Pathology, Lancet Laboratories and BARC-SA, Johannesburg, South Africa; 12 Institut National de Recherche Biomedicales, Kinshasa, Democratic Republic of Congo and Service de Microbiologie, Cliniques Universitaires de Kinshasa, Kinshasa, Democratic Republic of Congo; 13 AIDS national laboratory and Service de Microbiologie, Cliniques Universitaires de Kinshasa, Kinshasa, Democratic Republic of Congo; 14 Departments of Biomathematics and Human Genetics David Geffen School of Medicine at UCLA, and Department of Biostatistics UCLA School of Public Health, Los Angeles, United States of America; 15 Institute of Human Virology, Abuja, Nigeria; 16 KU Leuven, Department of Microbiology and Immunology, Rega Institute, Laboratory for Clinical and Epidemiological Virology, Leuven, Belgium; 17 Spatial Epidemiology Lab, Université Libre de Bruxelles, Bruxels, Belgium; University of North Carolina at Chapel Hill, UNITED STATES

## Abstract

Since the ignition of the HIV-1 group M pandemic in the beginning of the 20th century, group M lineages have spread heterogeneously throughout the world. Subtype C spread rapidly through sub-Saharan Africa and is currently the dominant HIV lineage worldwide. Yet the epidemiological and evolutionary circumstances that contributed to its epidemiological expansion remain poorly understood. Here, we analyse 346 novel *pol* sequences from the DRC to compare the evolutionary dynamics of the main HIV-1 lineages, subtypes A1, C and D. Our results place the origins of subtype C in the 1950s in Mbuji-Mayi, the mining city of southern DRC, while subtypes A1 and D emerged in the capital city of Kinshasa, and subtypes H and J in the less accessible port city of Matadi. Following a 15-year period of local transmission in southern DRC, we find that subtype C spread at least three-fold faster than other subtypes circulating in Central and East Africa. In conclusion, our results shed light on the origins of HIV-1 main lineages and suggest that socio-historical rather than evolutionary factors may have determined the epidemiological fate of subtype C in sub-Saharan Africa.

## Author summary

Since it emerged in human populations in the Democratic Republic of Congo (DRC) around 1920, HIV diversified in several virus lineages that spread across sub-Saharan Africa and beyond. While some lineages are rare and remain geographically confined to certain regions, e.g. subtypes H and J, others expanded rapidly across sub-Saharan Africa, e.g. subtype C. Here we conducted a spatial genetic analysis of the three main HIV-1 virus lineages, subtypes, A1, C and D, that co-circulate across 20 locations in central and East Africa, to investigate their spatial and temporal origins and their mode of spread across comparable geographic areas. We find that subtype C, currently the dominant lineage in sub-Saharan Africa, emerged in the southern DRC mining region, and spread 3-fold faster than other co-circulating lineages. Our study uncovers distinct patterns of spread of the main HIV-1 subtypes in a region that covers nearly half of all infected individuals in sub-Saharan Africa.

## Introduction

AIDS is one of the most devastating infectious diseases in human history and its main causative agent, HIV-1 group M, is responsible for over 38 million infections [1]. Several lines of evidence indicate that group M originated in Kinshasa, the capital city of the Democratic Republic of Congo (DRC), during the early twentieth century [2–5]. From there, founder events introduced virus lineages to other geographic regions [6], resulting in today’s heterogeneous global distribution of the genetic forms of HIV-1 group M, characterised by 9 subtypes and many recombinant forms [7]. Some of these lineages, such as subtypes H and J, are rare and remain largely confined to Central Africa [8–10]. Others are circulating in relatively more restricted regions, like subtype D in Central Africa [11]. In sharp contrast, subtype C expanded rapidly across sub-Saharan Africa, especially eastern and southern Africa, and is responsible for over 75% of HIV-1 cases in the region [12]. Overall, subtype C is currently the dominant HIV lineage worldwide and is responsible for nearly half of the world’s HIV infections [13].

HIV-1 cases in eastern and southern Africa comprise half of the HIV-1 infection burden worldwide [1]. In these regions, interconnectivity among population centres [11, 14], often facilitated by labour migration [15], together with the rapid growth of urban centres [16], and socio-historical changes [17, 18] are thought to have contributed to the spread and establishment of HIV-1. Subtype C is the most dominant in the region, followed by subtypes A1 and D [12]. It has been proposed that the dominance of subtype C in sub-Saharan Africa is the result of its increased transmission efficiency compared to other HIV-1 subtypes [19]. Subtype C incidence in Kinshasa has increased nearly 5-fold between 1997 and 2002 [20]. While the timing of subtype C emergence is well resolved [2, 21], the spatial origins and the ecological circumstances that drove its dominance in the region remains poorly understood. This is partly due to the limited amount of HIV-1 genetic data available from the DRC [11, 22–24], the epicentre of HIV-1 group M pandemic [2].

In this study, we investigate the spatial origins of the main HIV-1 subtypes using 346 newly-generated protease and reverse transcriptase sequences from the DRC. We undertake a comparative genetic analysis of the three main HIV-1 subtypes, A1, C and D, that have persistently co-circulated across 20 locations in Burundi, DRC, Kenya, Rwanda, Tanzania and Uganda and we elucidate their evolutionary dynamics and patterns of spatial spread. Our analysis extends our understanding of the origins of the subtype C epidemic, and reveals distinct patterns of spread of the main HIV-1 subtypes in a region that covers ~40% of all infected individuals in sub-Saharan Africa.

## Results

### Large-scale phylogeographic trends of HIV-1 group M

We investigate the spatial origins of HIV-1 subtypes in the DRC using reverse transcriptase and protease coding regions sequenced from 346 strains sampled in 2008 from several locations in the country: the capital city of Kinshasa (*n* = 80), the port western city of Matadi (*n* = 114) and the southern cities of Mbuji-Mayi (*n* = 85) and Lubumbashi (*n* = 67) (Table 1, S1 Fig; see the Materials and Methods section). To avoid impact of potential sampling biases on ancestral reconstruction [25], discrete trait ancestral reconstructions were performed using three data sets with the same number of taxa from Kinshasa, Matadi and Mbuji-Mayi (*n* = 80).

**Table 1:** Characteristics of the data sets and estimated evolutionary parameters. N: number of sequences per discrete location/country (*k*) in the DRC and in Central and East Africa (CEA). Δt = time interval of sequence sampling in this study. *ρ* indicates the Pearson’s correlation coefficient between N and HIV prevalence in 2013 for Burundi, DRC, Kenya, Rwanda, Tanzania and Uganda as recorded by UNAIDS [113]. P-values indicate statistical significance under a linear regression parametric model. The cumulative number of sequences per country and HIV seroprevalence over time can be found in S5 Fig. R^2^ indicates the correlation between genetic divergence and sampling dates (S6 Fig).

Characteristics	Subtype C	Subtype A1	Subtype D
N_DRC_ (*k*), N_CEA_ (*k*)	91 (4), 304 (20)	68 (4), 504 (20)	15 (4), 447 (20)
Δt (years) _CEA_	1997–2011.03	1996–2011.13	1996–2010.88
*ρ* (N_CEA_, Prev) (*p*-value)	0.47 (0.079)	0.71 (0.022)	0.19 (0.21)
R^2^ (root-to-tip) _CEA_	0.016	0.080	0.079

**Fig 1:**
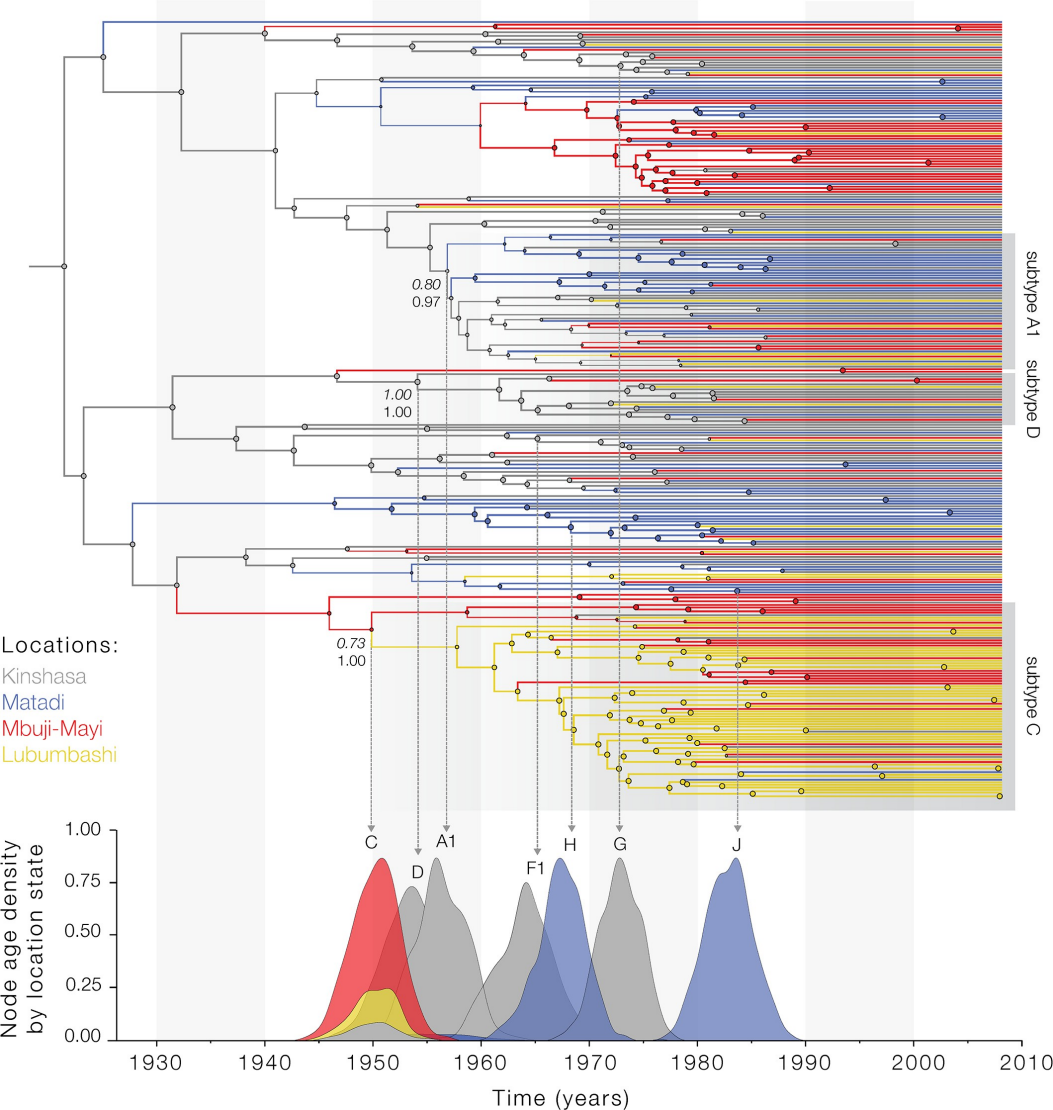
Origins and early spread of HIV-1 virus subtypes in the Democratic Republic of Congo (DRC). Maximum clade credibility tree based on 346 *pol* sequences sampled in the DRC in 2008. Branch colours depict the most probable inferred ancestral location as inferred using discrete phylogeographic analysis [26]. Grey boxes indicate the positions of isolates identified as belonging to subtype A1, C and D both by REGAv3.0 [71] and COMET [72] subtyping tools. Mean posterior support for the modal location estimates (upper italic number) and phylogenetic support values (lower number) are shown for specific viral lineages and for the root location. Since the DRC *pol* sequence alignment did not contain temporal information (all sequences were sampled in 2008), subtype node heights were calibrated using information from a previously published molecular clock analysis of *env* sequences [2]. The lower panel shows the node age density distributions stratified by location state for each subtype represented in this data set.

By jointly considering phylogenetic and ancestral location uncertainty in a discrete phylogeographic framework [26], we consistently identify Kinshasa as the ancestral root location of group M diversity (mean location posterior probability, LPP, across the three data sets = 0.92, with a standard deviation across LPP estimates of 0.09; Fig 1, S1 Table). These results, obtained using isochronous *pol* sequences, confirm previous findings obtained through analysis of heterochronous *env* genes sequences with a different spatial coverage [2].

### Epidemic origins and early dispersal routes of major group M lineages

We next estimated the epidemic origins of the main HIV-1 subtypes in the DRC (Fig 1). Our results consistently place the common ancestor of subtype C in Mbuji-Mayi (LPP = 0.79, SD = 0.03; Fig 1, S1 Table), from where this lineage was introduced around the 1960s to the more southerly city of Lubumbashi in the province of Katanga, a region that borders Zambia where subtype C predominates [27]. In contrast, we estimate that the common ancestor of HIV-1 subtypes D, G, F1, and of sub-subtype A1 originated in Kinshasa (LPP = 0.91, SD = 0.01; Fig 1). Interestingly, our data indicate that the rare subtypes H and J originated in the less-connected coastal city of Matadi (LPP = 0.95, SD = 0.03 and LPP = 0.97, SD = 0.02 respectively; Fig 1). Overall, our findings suggest that subtype C first emerged in or around Mbuji-Mayi, before reaching and becoming established in Lubumbashi. This is consistent with Kinshasa and Lubumbashi being well-connected cities in the DRC with respect to historical railway infrastructure and volume of passengers [28] and with recent reports that reveal complex mosaic forms related to subtype C in the mining region [29]. In contrast, we find that subtypes A1, D and sub-subtype F1 emerged in Kinshasa, while the least common subtypes J and H seem to have most likely emerged in the poorly connected city of Matadi in western DRC.

### The spread of the main HIV-1 group M lineages to East Africa

To quantify the evolution and dispersal patterns of the dominant HIV-1 lineages out of the DRC and into East Africa, we collated additional HIV-1 genetic data from 20 cities and villages across Burundi, Kenya, Rwanda, Tanzania, and Uganda, collected between 1996 and 2011 (Tables 1 and S3). For comparative purposes, we focused here on central and East African (CEA) data sets of unambiguously typed subtype C, A1 and D sequences (see the Materials and Methods section). Together, these subtypes account for a total of >84% of all HIV-1 infections in East Africa [13]. All CEA data sets tested negative for inter- as well as intra-subtype recombination using the pairwise homoplasy index test (*p*-value > 0.95). Of note, maximum likelihood (ML) phylogenies with available sub-Saharan sequences and the DRC sequences reported here (subtype C = 5304, subtype A1 = 2187, subtype D = 1210) show that the DRC sequences are predominantly basal to all sub-Saharan diversity (S2–S4 Figs). The ML reconstructions are consistent with a model of limited migration events of each subtype into East Africa followed by rapid expansion in the region [11], and further support the origins of these subtypes in distinct DRC localities.

We initially reconstructed the epidemic histories of group M subtypes using a relaxed molecular clock model and a best-fitting non-parametric coalescent tree prior (path sampling and stepping stone model selection approaches consistently favour the skygrid model over several parametric coalescent models, for all subtypes; S4 Table). Because we find a significant tendency for CEA sequences to cluster according to location of sampling (*P* < 0.001; S5–S7 Tables), we next investigated the spatial patterns of virus spread using an approach that combines information from multiple sources. Specifically, we used a hierarchical discrete phylogeographic model that shares a migration graph across subtypes while allowing some variability in the migration patterns at the subtype level [26, 30]. Although inference of location of the root of subtype C in Mbuji-Mayi was robust to the inclusion of East African data, the same was not true for the subtype A1 and D phylogenies, because of a much larger sample of East African sequences compared to the number of DRC sequences available for analysis. To correct for this bias, the root location of subtype A1 and D phylogenies was constrained to Kinshasa, as supported by our DRC-only analysis (Fig 1) and reconstructions of larger sub-Saharan Africa data sets (S2–S4 Figs).

**Fig 2:**
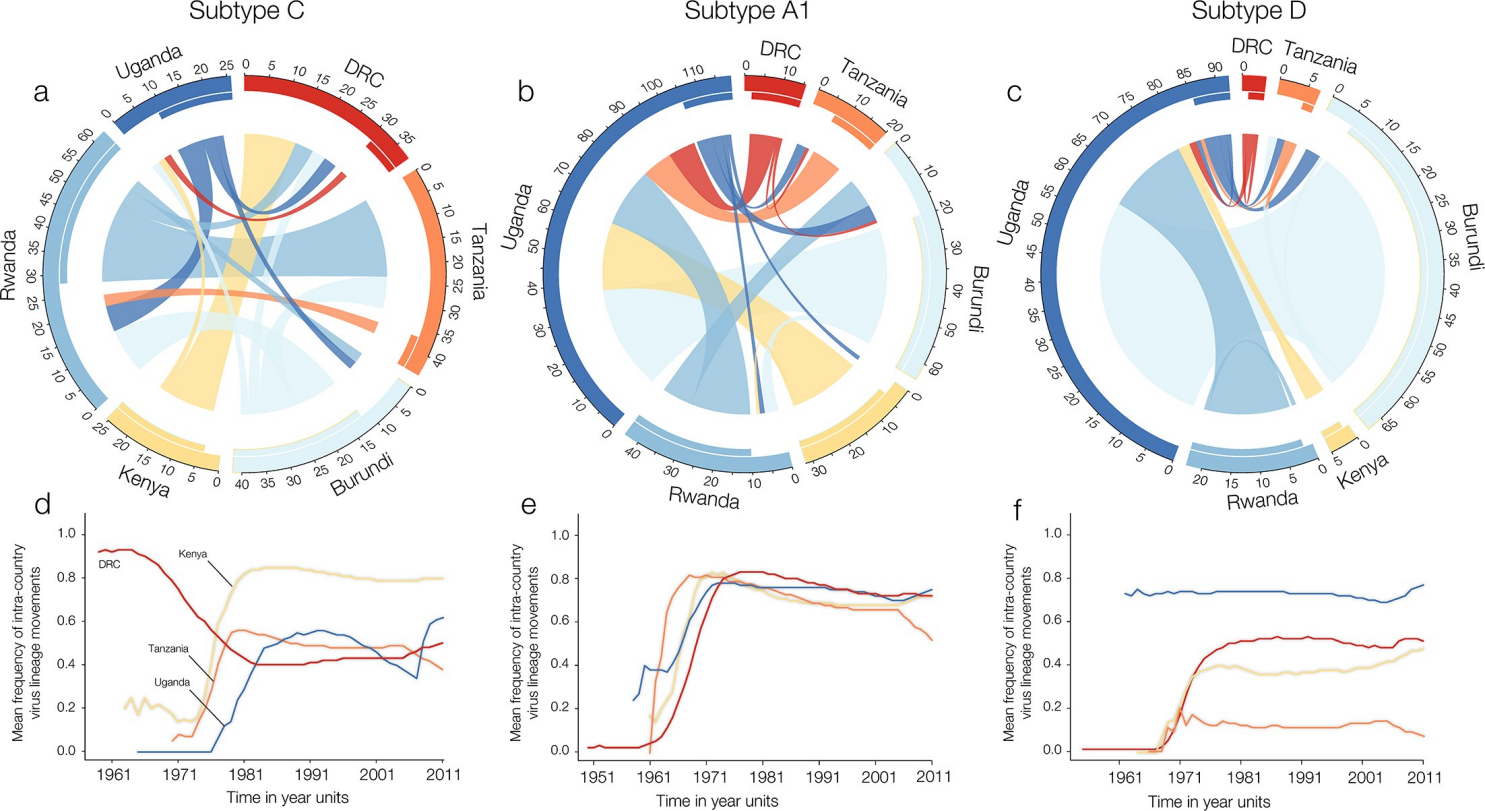
Pathways of HIV-1 lineage movement across Central and East countries. Fig 2A, 2B and 2C summarise lineage migration estimates for subtypes C-A1-D across Central East Africa and are represented using circular plots. Origin and destination locations of virus lineage movements are connected by circle segments. The width of the link at its basis indicates the frequency of viral movements as estimated using a robust counting approach and can be interpreted using the tick marks on the outside of the circle’s segments. The directionality of the virus lineage movement is encoded by the origin colour and by the gap between link and circle segment at the source location. Fig 2D, 2E and 2F show the estimated proportion of virus lineage movements through time within sampled countries with more than one sampling location. A proportion equal to 1 would indicate that all inferred movements occurred within a single country. This analysis only included virus lineage migration movements between countries for which data were available for at least two locations.

Our phylogeographic analyses of the main HIV-1 subtypes in Central and East Africa reveal distinct patterns of virus spread across the region. Fig 2 summarises the dispersal of HIV-1 subtypes across six Central and East African countries using circular plots [31–33]. These plots depict the estimated virus lineage movement among different countries in Central and East Africa for subtypes C, A and D (Fig 2A–2C). The absolute difference between the outer (migrations from-) and the inner (migrations into-) links reflect net migration for each country and subtype. Moreover, these results suggest that Rwanda, DRC and Tanzania have been the main net exporters of subtype C in the CEA region, while Uganda appears to act as the main source of subtypes A1 and D (Fig 2B and 2C).

We also estimated the proportion of within-country virus lineage movement through time by analysing ad hoc posterior estimates of the inferred transitions between countries [32–34] (Fig 2D–2F). Following a 15-year period of circulation in southern DRC, we found that subtype C was introduced in Kenya, Tanzania and Uganda around the 1970s (Fig 2D). Across-border virus lineage movement was stabilised around 1980 for subtype C (Fig 2D). Subtype A1 became established in East Africa from the 1970s onwards [11]. Following this, around 75% of estimated virus lineage movement occurred within the countries of sampling (Fig 2E). The patterns observed for subtype D suggest an early founder-effect from the DRC (perhaps indirectly through other Central African countries) to Uganda around 1960 (Fig 2F).

To quantify the contribution of virus movement across locations we used a hierarchical phylogeographic approach that estimates which migration rates between locations are likely to be relevant [30]. We find that on average 60% of the total number of estimated virus lineage movement events (38 of 64 links supported by Bayes factor, BF > 10) occurred within national borders (S8–S11 Tables). We calculate that on average 80% of all well-supported virus movements occurred at the within-country level (8 out of 10 total links supported by BF > 10; S11 Table) in comparison to cross-border movements. Taken together, these estimates suggest that approximately 20% of viral lineage movements result from cross-border transmissions.

### Faster spatial spread of subtype C compared to subtypes A1 and D

**Fig 3:**
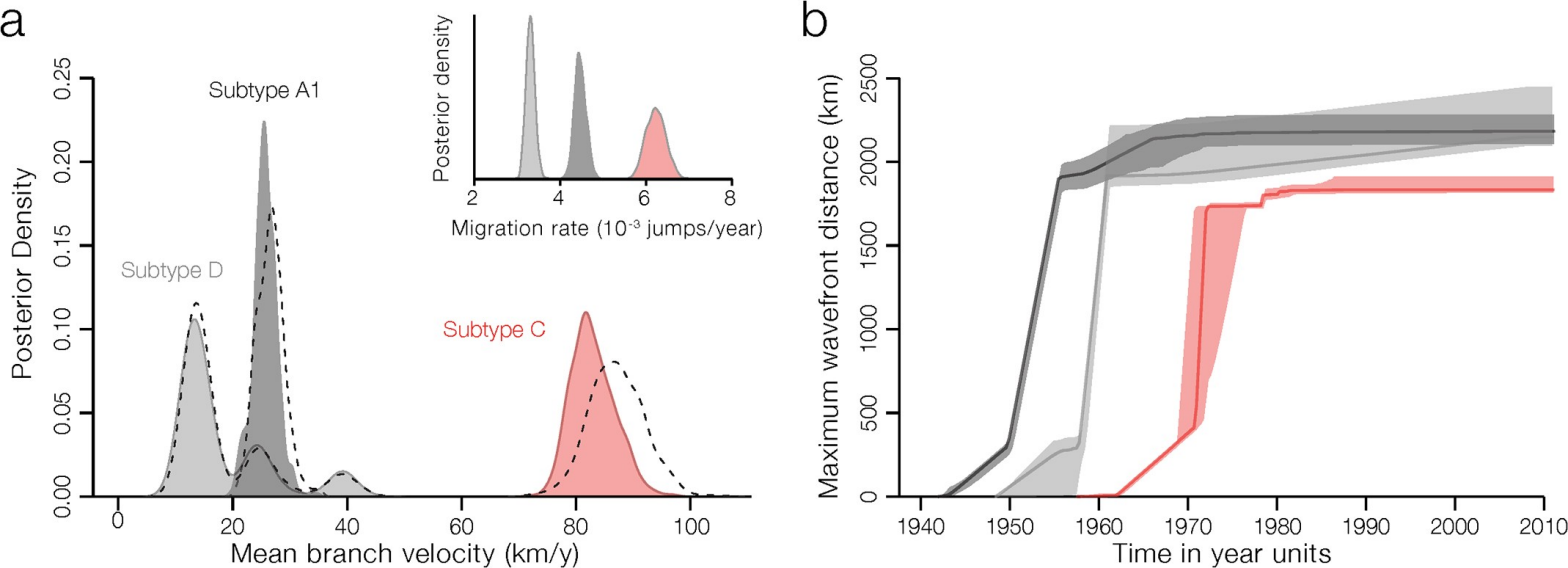
Velocity of spread of main HIV-1 lineages in Central and East Africa. (**a**) Posterior estimates for the branch dispersal velocity of HIV-1 subtypes. Filled and non-filled distributions represent respectively estimates from analyses with and without informed root location priors (see Materials and Methods for details). The inset shows the number of location transitions per time unit obtained using a robust counting approach. (**b**) Change of the epidemic wavefront through time, measured as the furthest extent of the subtype’s inferred location of origin to inferred branch locations.

To estimate the velocity of spatial spread of the different viral lineages, we used a phylogenetic model of diffusion in continuous space [35, 36]. This approach infers latitude and longitude locations for ancestral nodes in a phylogeny using a relaxed random walk model. When projected through space and time, the reconstruction of the subtype C spatial diffusion further supports a spatial origin in or around Mbuji-Mayi and reveals a rapid eastward spread of subtype C (S7 Fig). By 1980, subtype C had reached all African countries under investigation. Notably, we find that the lineage dispersal velocity of subtype C was three-fold higher compared to subtype A1 and four-fold higher compared to subtype D (Fig 3A, Table 2). These estimates are robust to using informative or uninformative of root location priors (Fig 3A). The faster lineage dispersal velocity for subtype C is also confirmed by Markov jump count estimates of the location state transitions in a discrete phylogeographic approach [32, 37].

**Table 2:** Geographic origins and rate of spread of HIV-1 in Central and East Africa. Posterior probability for the ancestral root location in the DRC (details in S1 Table). CEA: Central East Africa. BCI: Bayesian credible interval. Mean branch dispersal velocities and mean diffusion coefficients were estimated using the R package “seraphim” [111].

Posterior estimates	Subtype C	Subtype A1	Subtype D
Root location in DRC (posterior probability)	Mbuji-Mayi (0.91)	Kinshasa (1.00)	Kinshasa (0.99)
Migration rate in DRC (10^−3^ jumps/year, 95% BCI)	15.33 [13.01–17.68]	10.03 [8.59–13.22]	9.92 [0.01–14.42]
Migration Rate in CEA (10^−3^ jumps/year, 95% BCI)	14.1 [11.79–15.91]	8.65 [7.75–9.69]	6.03 [5.60–6.46]
Mean branch velocity in CEA (95% BCI) (km/year)	82.43 [[76.80–90.99]	25.68 [21.31–30.08]	14.41 [11.30–40.36]
Mean diffusion coefficient *D* (km^2^/year)	19562.55 [16873.86–21910.36]	3450.91 [2783.84–4374.69]	1283.41 [1004.30–15898.77]

Our subtype C analysis reveals rapid movement of the epidemic wavefront until early 1980s, after which the maximal extent of epidemic spread had been reached (Fig 3B). For subtypes A1 and D, the epidemic wavefront expanded from its origin somewhat earlier (Fig 3B), in agreement with a scenario of early long-distance movements seeding these lineages into East Africa.

### No evidence for distinct transmission rates among HIV-1 subtypes

In an attempt to disentangle why subtype C is more prevalent than subtypes A1 and D, we sought to infer its transmission potential directly from sequence data [38]. Several studies have suggested a higher transmissibility of subtype C compared to A [39], and a higher transmissibility of subtype A1 compared to D [40]. This could explain the increase in the prevalence of subtype C in Kinshasa from 2.1% in 1997 to 9.7% in 2002 [20], and to 15% in 2007–2008 (see also S1 Fig). To investigate the transmission potential of subtype C we consider the subtypes’ basic reproductive number R_0_, which is defined as the number of secondary infections that arise from a typical primary case in a completely susceptible population. Assuming a sampling probability that takes into account the number of isolates per subtype in relation to the number of infected people in each country [41], our genetic estimates obtained using a birth-death model indicate an R_0_ ~ 3 for all subtypes, with no statistically significant differences among subtypes (S12 Table). However, as reported in S12 Table, credible intervals associated with R_0_ estimates are relatively large and could potentially prevent the detection of an actual difference among subtypes. Overall, these estimates are in line with previous findings [38, 42], but relatively lower than some estimates obtained with an alternative logistic coalescent model specifically focusing on sub-clades of subtype C (R_0_ ~ 5–6). For our analyses, we however favoured the use of a birth-death model, mainly because it has been demonstrated that birth-death models deliver better performances when approximating stochastic exponential population growth [43].

Finally, genetic data allow us to estimate the rate of each subtype’s exponential growth (*r*). This rate can be directly comparable to R_0_ if the same duration of infection is assumed for all subtypes. Our results show that each subtype had a similar early epidemic growth rate, with median estimates of *r* between 0.43 to 0.52 per year (S12 Table) and widely overlapping uncertainty intervals. Therefore, our genetic analyses provide no evidence for different transmission potentials of subtypes A1, C and D.

### Evolutionary rates and selection pressure in the pol gene of the main lineages circulating in Central and East Africa

It has been hypothesised that subtype C may have a selective advantage in comparison with other co-circulating strains [44]. As part of our Bayesian framework, we estimate selective pressure as the ratio of non-synonymous substitutions over synonymous substitutions (dN/dS) for each subtype while integrating over the posterior distribution of phylogenies, in order to quantify uncertainty in nonsynonymous and synonymous substitution estimates [31]. While synonymous rates are expected to reflect mutation rates and generation times, non-synonymous rates will also be affected by (immune) selective pressure.

**Fig 4:**
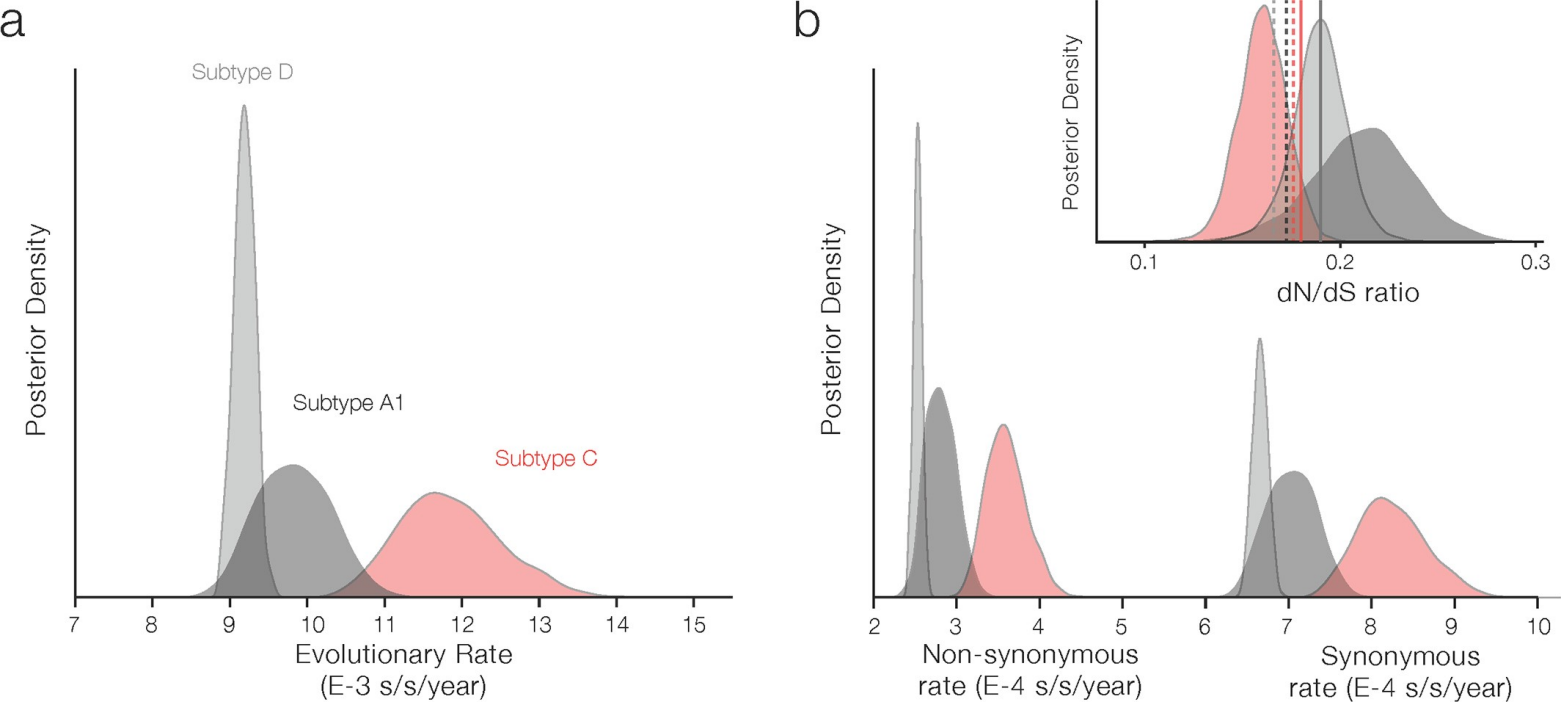
Synonymous and non-synonymous rates of substitution for main subtypes circulating in Central and East Africa. **Panel a** shows the posterior distributions for the overall nucleotide substitution rates (substitutions per nucleotide site per year) based on the entire gene region under study. **Panel b** shows the absolute non-synonymous and the synonymous rates obtained using a renaissance counting approach. Finally, the inset shows the dN/dS ratio obtained using a renaissance counting approach [31], with dashed and solid lines indicating the mean dN/dS ratios estimated with MEME [98] and aBSREL [99]. Please note that the mean dN/dS ratios for subtypes A1 (dark grey solid line) and D (light grey solid line) estimated with aBSREL are overlapping.

Interestingly, we find a higher synonymous rate for subtype C compared to A1 and D, and similar but less pronounced differences in nonsynonymous rates (Fig 4B). Our analysis shows that the *pol* region is subjected to strong overall purifying selection [45] with a posterior mean of dN/dS ratios of 0.18 across subtypes (Fig 4B). These ratios, obtained using a Bayesian renaissance counting approach, are consistent with point estimates using simpler methods that provide per-site dN/dS ratios (Fig 4B, dashed and solid vertical lines). Given that these estimates for dN/dS ratios indicate that most residues are under strong negative selection, such averaging can also mask strong heterogeneity in positive selection acting upon sites along the gene. Consequently, although these findings suggest the *pol* gene in subtype C lineage has not experienced strong positive selection during its evolutionary history, we cannot eliminate the possibility that positive selection may occurred at some sites or outside the *pol* region under study that may have led to an increase in transmission potential.

## Discussion

Whether the rapid expansion of HIV-1 subtype C has been the result of ecological or evolutionary circumstances has been a matter of debate. We investigated the evolutionary origins of the HIV-1 group M diversity by analysing *pol* data from the DRC with data from eastern Africa. We find that the emergence of HIV-1 subtypes resulted from two distinct scenarios (i) where ancestral lineages from Kinshasa seeded epidemics elsewhere, e.g., subtype C in Mbuji-Mayi and subtype H in Matadi, and (ii) the emergence and circulation of subtypes within Kinshasa such as sub-subtype A1, and subtypes D, G and F1 (Fig 1). For scenario (i), two contrasting epidemiological outcomes seem to occur, depending on the remoteness/accessibility of the location where different lineages first emerged. For example Mbuji-Mayi, where subtype C emerged, was a thriving, well-connected, economic hub with the largest production of diamonds in the world, which attracted migrants from Lubumbashi and from other countries, such as Zambia and Zimbabwe [28]. In contrast, Matadi, a remote port city in Central west DRC, where subtype H and J are estimated to have emerged, was relatively poorly connected to the rest of the sub-Saharan African region. As a result, transmission of subtypes H and J is now confined to Central west DRC and neighbouring areas in northern Angola [8, 9, 46]. In contrast, scenario (ii) is consistent with local circulation of group M strains in Kinshasa that may have been associated with different transmission routes and/or different risk groups since the beginning of the HIV pandemic [41]. It is possible that some of these lineages became extinct, e.g. subtype E, while others may have been amplified by iatrogenic transmission in Kinshasa between 1950–1960 [2], similar to the spread of hepatitis C virus in Kinshasa [47].

The results presented here further reveal that subtype C most likely emerged in the mining city of Mbuji-Mayi, and spread significantly faster than subtype A1 and D throughout Central and East Africa. The spatial origins in Mbuji-Mayi are consistent with recent reports suggesting complex mosaic forms related to subtype C in this city [29]. These results further suggest that the earliest dispersal events of subtype C (S7 Fig) occurred in a mining region [28] that includes Mbuji-Mayi (e.g. diamond mining; Kasai region) and Lubumbashi (e.g. Copperbelt; Katanga). The latter is the largest city in southern DRC, and due to its railway connections to other African neighbouring countries, this region may have acted as a stepping stone for the larger epidemics in southern Africa. Railway networks in Uganda, Rwanda, Burundi, and Tanzania were among the earliest in sub-Saharan Africa [48]. In addition, truck drivers, who often travel long-distance and are known to be at a higher risk of HIV infection than the general population [49, 50], and populations living in the vicinity of highways, have also been shown to be at higher risk of acquiring HIV [51]. Road traffic around 1960 was highest in the northern highway connecting Mombasa to Kampala by Lake Victoria, and lower along the southern highway connecting Dar es Salaam to Mwanza, in southern Lake Victoria [48]. These connectivity patterns agree with the strongly supported virus lineage migration events we observe along the northern highway. Moreover, we estimated that the epidemic wavefront of subtype C travelled faster compared to other co-circulating lineages. These findings were robust to the type of model-based phylogeographic reconstruction. Our posterior estimates are directly comparable among subtypes because sequences were sampled across the same geographic range. However, we note that these estimates are dependent of the scale of sampling [36] and it may be more problematic to compare these spatial invasion rates to HIV-1 datasets collated from different geographic regions and sampling regimes.

Our study represents the largest survey of group M’s *pol* gene sequence diversity in the DRC and our results provide further support for an origin of group M in Kinshasa [2]. While previous analyses of partial *env* gene sequences have also placed the origin of group M in Kinshasa [2, 52], the recombinant nature of HIV implies that different genomic regions may have different evolutionary histories [53, 54]. More generally, our *pol* sequences from the DRC add detail to the origins of group M subtypes [11, 22, 55–57] and use a genomic region that is more commonly sequenced due to increased antiretroviral coverage. Therefore, the *pol* sequences will be useful for future genetic analysis at the country and continental-level.

Our hierarchical phylogeographic estimates indicate that pairs of locations that are closer to each other tend to be involved in more extensive virus exchange on average. The time to travel between locations has previously been suggested the shape the geographic distribution of human viruses [14, 58, 59] and railways have been suggested to play a role in the spread of HIV-1 in Central Africa [2]. Yet, the spatial distribution of subtype D most likely results from a single founder viral strain being introduced into Uganda. Although no subtype D *pol* data are available from the north of the DRC, *env* data indicate that subtype D is relatively prevalent in Bwamanda and Kisangani [20, 52], with the latter being the closest DRC city to Uganda. Once introduced to Uganda, HIV-1 rapidly became established in the country, most likely spreading within structured transmission networks [60]. Socio-historical factors may have played an important role in the establishment HCV epidemics in the DRC during the 1950s [47], so it is possible that similar factors in Uganda [61] may have facilitated the rapid expansion of subtype D in the country. With the advent of next generation sequencing and rapid generation of complete full genomes, we expect that data sets resulting from a larger and denser sampling will allow for a more detailed analysis of the historical and contemporaneous drivers of HIV-1 spread in Sub-Saharan Africa [62–64].

The data presented in this study indicate an increase in subtype C prevalence in Kinshasa from 2.1% in 1997 to 9.7% in 2002 [20] and 15% in 2007–2008 (S1 Fig). We detect higher synonymous substitution rates for subtype C that could in part be explained by a higher replicative capacity of subtype C compared to other lineages [65]. Higher overall subtype C substitution rates obtained by similar analysis of the gp41 region of *env* has recently described in a cohort of untreated Uganda patients [66]. The authors found that the nonsynonymous substitution rate in the gp41 region of *env* is twice as fast for subtype C compared to other subtypes [66].

Overall, we find no evidence of a strong selective advantage nor for increased transmission potential for subtype C. We here postulate that founder effects and epidemiological circumstances, such as introduction to and circulation among mining populations in the southern DRC with subsequent onward spread through the Copperbelt mining region, are more likely to have contributed to the success and spatial spread of subtype C throughout sub-Saharan Africa, where it currently accounts for 75% of HIV infections. However, we acknowledge that the estimates presented here relate only to the polymerase region of the virus genome and we contend that future studies that generate large full-genome data sets from sub-Saharan Africa will allow to investigate lineage-specific determinants of between-host and within-host transmission. Future work could improve on this study by performing comparative, genome-wide analyses of HIV-1 subtypes [67], and by adding finer scale epidemiology to identify HIV hotspots [68, 69]. Our results show that the complex patterns of HIV epidemics have been shaped by migration and travel and therefore it is important to consider both local and international strategies when designing interventions to end HIV transmission in sub-Saharan Africa.

## Materials and methods

### Sequencing of HIV-1 isolates

Protease and reverse transcriptase were obtained for 346 patients attending antiretroviral therapy clinics and public hospitals in 2008 in four cities across the Democratic Republic of Congo, Kinshasa, Matadi, Mbuji-Mayi and Lubumbashi, during previously reported surveillance studies on drug resistance in the DRC [70]. In brief, viral RNA was extracted from the plasma using the QIAamp Viral RNA kit (Qiagen, Courtaboeuf, France). RNA was transcribed into cDNA with the reverse primer IN3, cDNA was amplified by a nested polymerase chain reaction using the Expand High Fidelity PCR system (Roche, Meylan, France) with outer primers G25REVand IN3 and inner primers AV150 and polM4. The amplified fragments covering the protease (amino acids 1–99) and reverse transcriptase (amino acids 1–310) were purified with the QIAquick Gel Extraction kits (Qiagen) and directly sequenced using the BigDye Terminator v3.1 Cycle Sequencing kit (Applied Biosystems, Carlsbad, CA). Sequences were assembled with the SeqMan II software (DNASTAR, Madison, WI).

### Collation of HIV-1 genetic data sets

Sequences were subtyped using REGAv3.0 [71] and COMET [72]. A further 96 sequences sampled in 2007 from military personnel serving in Kinshasa were included for analysis [73]. Data with concordant subtype assignments and pertaining to the three most commonly detected lineages in the DRC, i.e. sub-subtype A1 (*n* = 84), subtype C (*n* = 92) and subtype D (*n* = 15) were retained for subsequent analysis. We then compiled 2,960 sequences collected from 1996 to 2011 across 16 locations (cities or villages) spanning 5 East African countries with generalized epidemics, namely Burundi [74, 75], Kenya [76–78], Rwanda [79], Tanzania [80–82] and Uganda [83–88] (sampling locations are displayed in S7 Fig). DRC and East African sub-subtype A1, subtype C and subtype D data sets were then merged to form Central East African (CEA) data sets (*n* = 1,916; mean, minimum and maximum of 99, 44 and 170 sequences per location, respectively). Major resistance-conferring sites at the amino acid positions described by the International AIDS Society–USA were excluded for phylogenetic analysis [89]. In order to mitigate potential sampling bias in these data sets, we randomly sub-sampled sequences so that the number of taxa from each location across subtypes was roughly proportional to the estimated HIV-1 prevalence in each corresponding country (multiple *R*^*2*^ = 0.69, p-value 0.025). S3 Table shows the number of sequences per location between and after subsampling. To contextualise these data, sub-Saharan African overlapping sequences deposited on the LANL-HIVdb [7] belonging to subtype A1 (*n =* 2,187), subtype C (*n =* 5,304) and subtype D (*n =* 1,210) (accessed May 2015, LANL) were appended to the CEA data sets.

### Estimating temporal and spatial signal

Multiple sequence alignment was performed using MAFFT v7 [90] and manually curated using Se-Al (http://tree.bio.ed.ac.uk/software/seal). Maximum likelihood (ML) phylogenies were reconstructed in FastTree v.2 using the GTR+4Г nucleotide substitution model [91]. A regression analysis [92] was used to determine the correlation between sampling dates and divergence to the root of midpoint rooted maximum likelihood (ML) CEA phylogenies (S6 Fig). To assess whether virus populations were structured per country compartmentalization analyses were performed using tree-based methods such as the association index estimated using a posterior distribution of phylogenies in BaTS [93] and estimated using maximum likelihood Simmond’s Association Index (AI) [94] implemented in HyPhy [95]. When sequences are labelled according to location of sampling, the two statistics strongly reject the null hypothesis of panmixis (S2 Table; observed AI = 19.6, expected AI under panmixis = 28.6, *P* < 0.001; observed PS = 162.6; expected PS under panmixis = 231.3, *P* < 0.001).

### Checking for recombination

To check for inter- and intra-subtype recombination, we applied the Φ-test [96] implemented in the program SplitsTree 4 [97]. The Φ-test is based on a pairwise homoplasy index (PHI), which is a measure of the similarity between closely linked sites. In the test, the level of significance of this statistic is tested by permuting the sites. The rationale behind this permutation procedure is that under the null hypothesis of no recombination, the genealogical correlation between sites is not altered by such permutations because all sites are linked and share the same evolutionary history [96].

### Selection analysis

The posterior trees for the different subtypes from the above analysis were used as empirical tree distributions for estimating evolutionary rates (nonsynonymous and synonymous) and dN/dS ratios using the renaissance counting method [31] implemented in BEAST 1.8.4. Two independent MCMC runs of 10 million steps were computed for this analysis using BEAST. We also estimated dN/dS ratios for the three main subtypes in HyPhy [95] with two maximum-likelihood based methods; MEME [98] and aBSREL [99], which provide site-specific dN/dS ratios using a mixed effects site model and an adaptive branch-site random effects model, respectively. Both MEME and aBSREL relax the assumption that selective pressure at a site and/or branch is constant across the phylogeny [98, 99].

### Reconstruction of time-scaled phylogenies

To reconstruct the evolutionary history of HIV-1 lineages, we used Bayesian inference through a Markov chain Monte Carlo (MCMC) framework as implemented in BEAST 1.8.4 [100], and BEAGLE library 2.1.2 [101] to increase computational performance. For each subtype, mid-point rooted ML trees were used as starting trees. We employed the GTR+4Г [102] and an uncorrelated relaxed molecular clock model with an underlying lognormal distribution [103]. Since little or no temporal signal was present in our data sets (Table 1, S6 Fig), normal priors were placed on the time of the most recent common ancestor (TMRCA) of sub-subtype A1, subtype C and subtype D based on an analysis of the genetic data from a previous study [2] (mean and 95% Bayesian Credible Intervals (BCIs) of the TMRCA’s used here are shown in S8 Fig). To ensure adequate mixing of model parameters, MCMC chains were run in triplicate for 250 million steps for each subtype, sampling 5,000 trees and 10,000 parameter estimates from the posterior distribution. The resulting MCMC chains were combined and inspected in Tracer 1.7 [98].

To identify the best-fitting coalescent model to describe changes in effective population size over time, model selection was performed using path-sampling and stepping-stone log-marginal likelihood estimators [104, 105]. Using the same amount of computational work (50 million path steps), distinct demographic models were tested: constant, exponential, exponential-logistic and the Bayesian skygrid with 60 grid-points [106]. Differences in log-marginal likelihoods are shown in S4 Table. After removal of 10–30% burn-in, subtype-specific empirical tree distributions (consisting of 1,000 time-calibrated trees) evenly sampled from the posterior distribution from the best-fitting model runs were generated for subsequent analyses.

### Counting within-country virus migrations through time

To perform ancestral reconstruction of the unobserved sampling countries (*k* = 6) and locations (*k* = 20), discrete phylogeographic analyses [26] were performed using the empirical tree distributions generated for the Central and East African data sets of subtypes C, A and D. To avoid over-parameterisation, for each subtype the location exchange process was modelled using symmetric continuous-time Markov chains [26] with an approximate CTMC conditional reference prior on the overall rate scalar and a uniform prior distribution [107]. Bayesian analysis was run using BEAST v1.8.4 [100] with BEAGLE library 2.1.2 [101] for an MCMC chain of 10 million iterations, sampling 10,000 samples of all parameters and 1,000 trees for each subtype. We jointly estimated the expected number of country and location changes along the branches of the posterior tree distribution using a “robust counting” approach implemented in BEAST v1.8.4 [32–34]. Specifically, we inferred on a branch-by-branch basis the history of virus movement between each pair of countries and each pair of locations. We used the R package “circlize” [108] to summarise the estimated number of migrations between countries in the form of circular plots. We also used an in-house script to estimate the proportion of within-country virus lineage movement over time. The script takes an input the “robust counting” files from BEAST v1.8.4, i.e. a tab-delimited file with three columns (source location, sink location, estimated date of virus migration), and a tab delimited file containing 2 columns (location, country), and generates the proportion of virus lineage movement within a single country over time (available from the authors upon request).

### Identifying pathways of virus spread using graph hierarchies

To identify a subset of well-supported migration events amongst subtypes we use a Bayesian Stochastic Search variable selection procedure (BSSVS) with a hierarchical prior on location and country indicators (0–1) that allow CTMC rates to shrink to zero with some probability [30]. Posterior distributions of country and location clock rates were obtained using separate conditional reference priors for each subtype [109]. Strongly supported rates of virus movement (Bayes factor > 10) were identified using SPREAD [110] and can be found in S8–S11 Tables.

### Spatial diffusion in continuous space

While ancestral locations inferred with the discrete approach will be necessarily drawn from the set of sampled locations and countries, we also use a relaxed random walk (RRW) model, in which diffusion rates were allowed to vary among branches according to a Cauchy distribution, to fully explore viral diffusion in the two-dimensional space (latitude and longitude) for the CEA data sets [36]. To sidestep a computationally demanding joint inference, we performed each continuous phylogeographic reconstruction on a single tree: the maximum clade credibility tree obtained using phylogenetic inference without ancestral state reconstruction, acknowledging that the inferred continuous diffusions do not accommodate phylogenetic uncertainty. For the sake of comparison, each continuous phylogeographic inference was performed with and without an informative prior on the root location, i.e. corresponding to the geographic coordinates associated with the inferred discrete location state for Kinshasa (subtypes A1 and D) Mbuji-Mayi (subtype C).

Subsequently, each phylogenetic branch connecting any two nodes was taken as an independent viral lineage movement event. The departure and arrival dates (*t*_*i*_, *t*_*f*_), and coordinates [(*x*_*i*_, *y*_*i*_), (*x*_*f*_, *y*_*f*_)] were then extracted from 1,000 trees resampled from the posterior distribution using the R package “seraphim” [111]. Three statistics were computed from this: (i) mean branch velocity (km/year), (ii) mean diffusion coefficient (km^2^/year), and the (iii) evolution of the maximal wavefront distance, i.e. distance in km from the estimated location of the root to each tip.

### Measuring transmission potential from genetic data

The basic reproductive number, R_0_, is defined as the number of secondary infections that arise from a typical primary case in a completely susceptible population. We opted for the birth-death model [38] available in BEAST v.2 software package [112] to quantify R_0_ based on time-stamped sequence data. Here we used a beta prior on the sampling probability parameter that takes into account the number of isolates per subtype in relation to the number of infected people in each country under analysis [41] (S12 Table). The birth-death model was here preferred over an alternative structured coalescent model because it has been demonstrated that the coalescent does not well approximate stochastic exponential population growth [43], which is typically modelled by a birth-death process. Finally, we used a constant-logistic growth model to estimate epidemic growth rates (*r*) during the exponential phase of the epidemic [2].

## Supporting information

S1 Table Ancestral location posterior probability (LPP) for group M and main subtypes for data sets 1 to 3. SD: standard deviation.
(XLSX)

S2 Table HIV-1 group M spatial admixture in the DRC. AI: association index, PS: parsimony score, CI: credible interval; P: statistical significance.
(XLSX)

S3 Table Characteristics of the Central and East African data set used for genetic analysis. (XLSX)

S4 Table Model selection result for the choice of coalescent tree prior. PS: path sampling, SS: stepping stone.
(XLSX)

S5 Table HIV-1 subtype C spatial admixture in Central and East Africa. AI: association index, PS: parsimony score, CI: credible interval; P: statistical significance.
(XLSX)

S6 Table HIV-1 subtype A1 spatial admixture in Central and East Africa. AI: association index, PS: parsimony score, CI: credible interval; P: statistical significance.
(XLSX)

S7 Table HIV-1 subtype D spatial admixture in Central and East Africa. AI: association index, PS: parsimony score, CI: credible interval, P: statistical significance.
(XLSX)

S8 Table Most significant pathways (Bayes Factor, BF, support < 10) of subtype C spread in Central and East Africa. (XLSX)

S9 Table Most significant pathways (Bayes Factor, BF, support < 10) of subtype A1 spread in Central and East Africa. (XLSX)

S10 Table Most significant pathways (Bayes Factor, BF, support < 10) of subtype D spread in Central and East Africa. (XLSX)

S11 Table Most significant pathways (Bayes Factor, BF, support < 10) of subtypes C, A and D spreads obtained with the hierarchical level approach. (XLSX)

S12 Table Epidemiological estimates for subtypes C, A and D in Central and East Africa. (XLSX)

S1 Fig Frequency of HIV genetic form sampled for each subtype and sampling location. (PDF)

S2 Fig Maximum likelihood phylogeny of subtype C based on publicly available sub-Saharan sequences and new sequences reported in this study. (PDF)

S3 Fig Maximum likelihood phylogeny of subtype A1 based on publicly available sub-Saharan sequences and new sequences reported in this study. (PDF)

S4 Fig Maximum likelihood phylogeny of subtype D based on publicly available sub-Saharan sequences and new sequences reported in this study. (PDF)

S5 Fig Cumulative numbers of sequences per country and HIV seroprevalence over time. (PDF)

S6 Fig Root-to-tip regression analyses of phylogenetic temporal signal. Correlation and determination coefficient (R^2^) were estimated with TempEst.
(PDF)

S7 Fig Spatiotemporal diffusion of HIV-1 subtypes A1, C and D across Central and East Africa. Internal nodes of maximum clade credibility and 95% HPD regions based on 1,000 trees subsampled from the posterior distribution of each continuous phylogeographic analysis. MCC tree internal nodes are coloured according to their time of occurrence, and 95% HPD regions were computed for successive time layers and then superimposed using the same colour scale reflecting time. Crosses indicate the position of the sampling locations.
(PDF)

S8 Fig Mean and 95% Bayesian Credible Intervals (BCIs) of the time of the most recent common ancestor (TMRCA) of each subtype. These values are used to define normal priors for TMRCA parameters estimated in BEAST analyses (see the text for further details).
(PDF)
