# Supplementary figures and images for: Distinct rates and patterns of spread of the major HIV-1 subtypes in Central and East Africa
Source: PLoS Pathog. 2019 Dec 6;15(12):e1007976. doi: 10.1371/journal.ppat.1007976 (PMC6897401; doi:10.1371/journal.ppat.1007976)

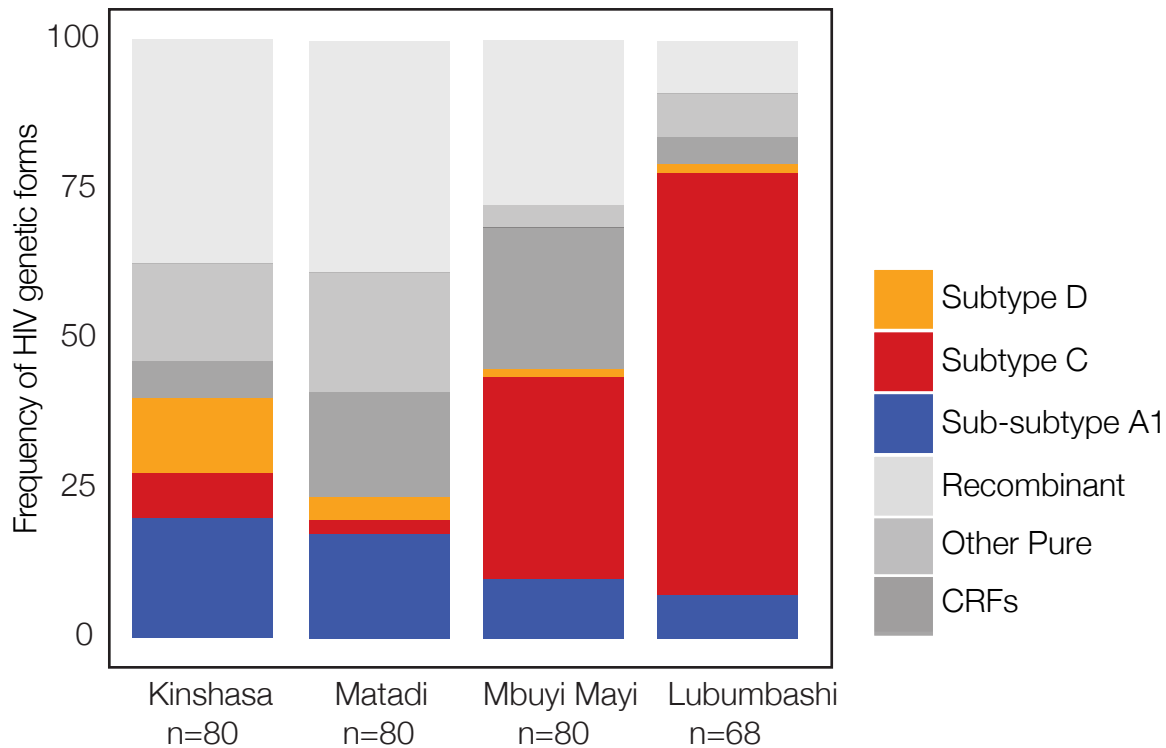

Supplement: S1 Fig — (PDF) [file ppat.1007976.s013.pdf]

DRC  
Middle Africa  
East Africa  
Western Africa  
Southern Africa

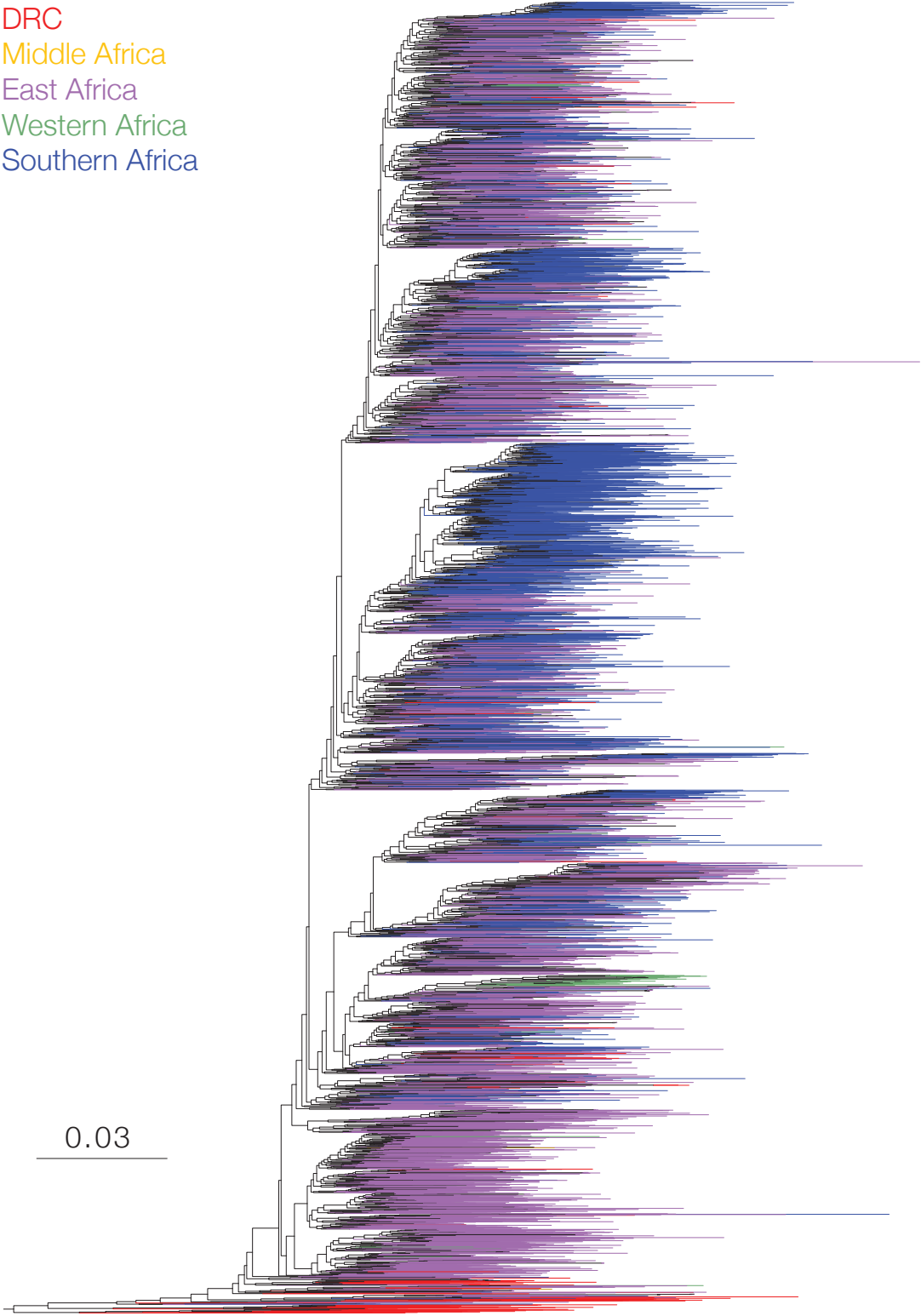

Supplement: S2 Fig — (PDF) [file ppat.1007976.s014.pdf]

DRC  
Middle Africa  
East Africa  
Western Africa  
Southern Africa

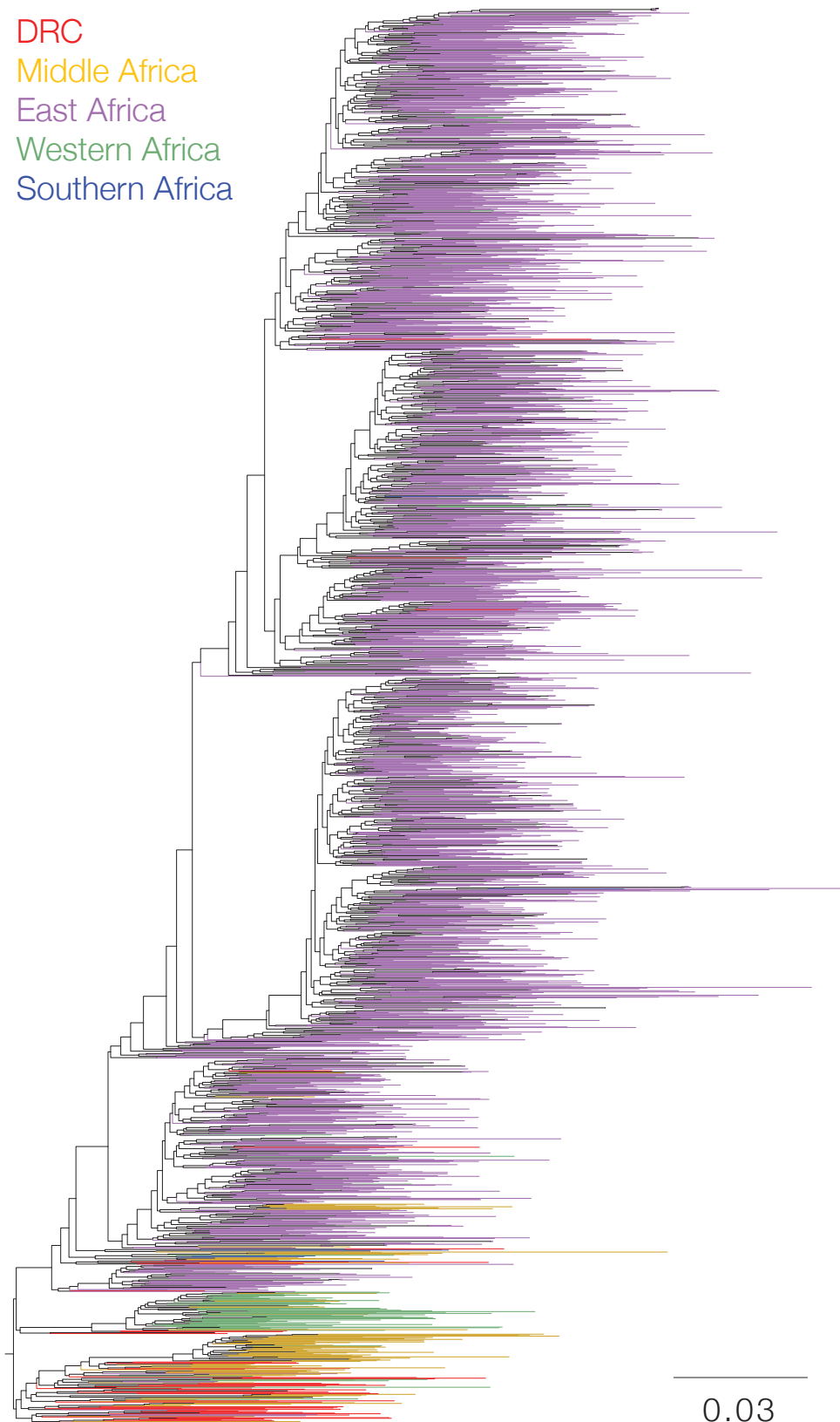

Supplement: S3 Fig — (PDF) [file ppat.1007976.s015.pdf]

DRC  
Middle Africa  
East Africa  
Western Africa  
Southern Africa

0.03

---

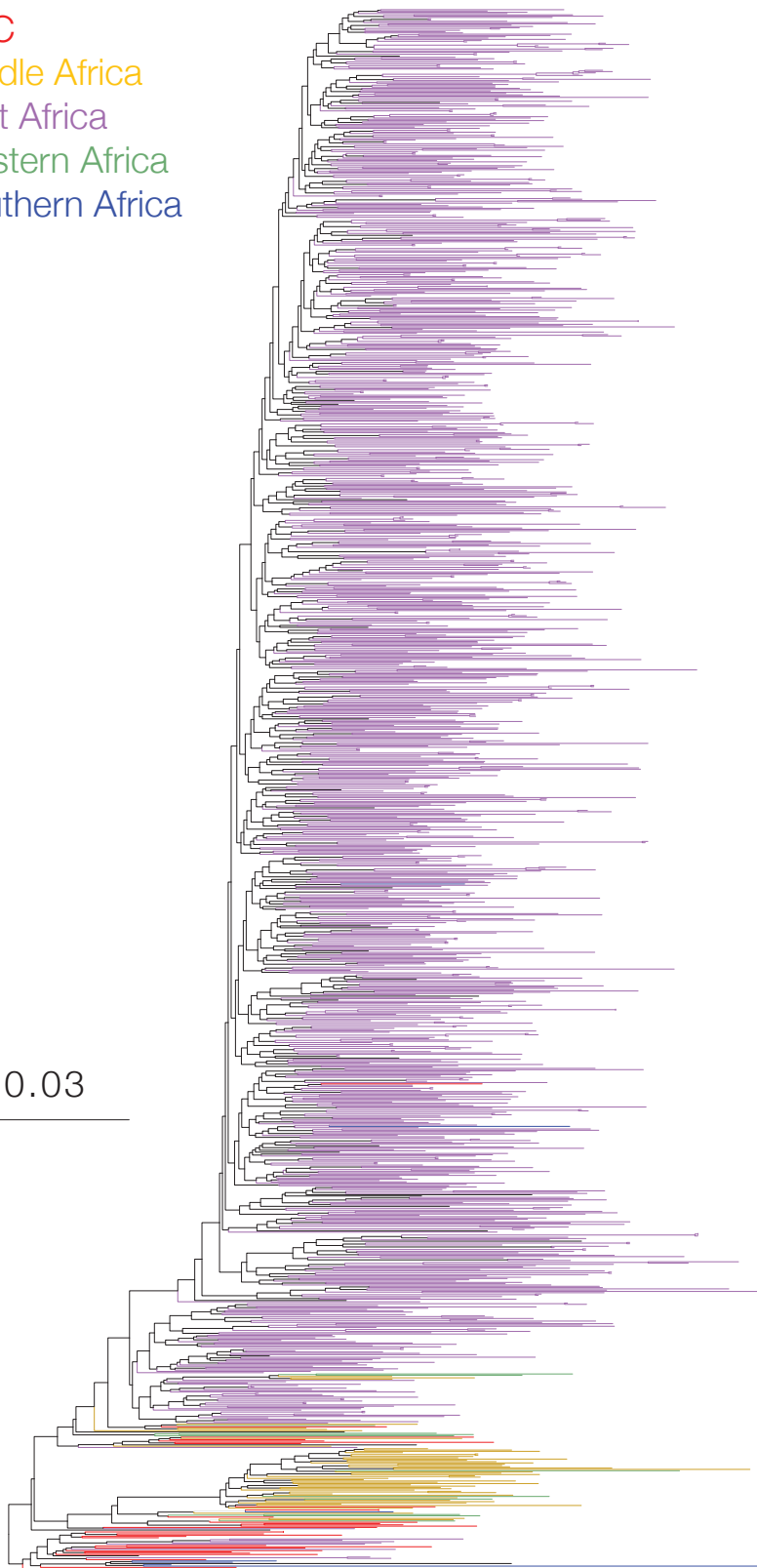

Supplement: S4 Fig — (PDF) [file ppat.1007976.s016.pdf]

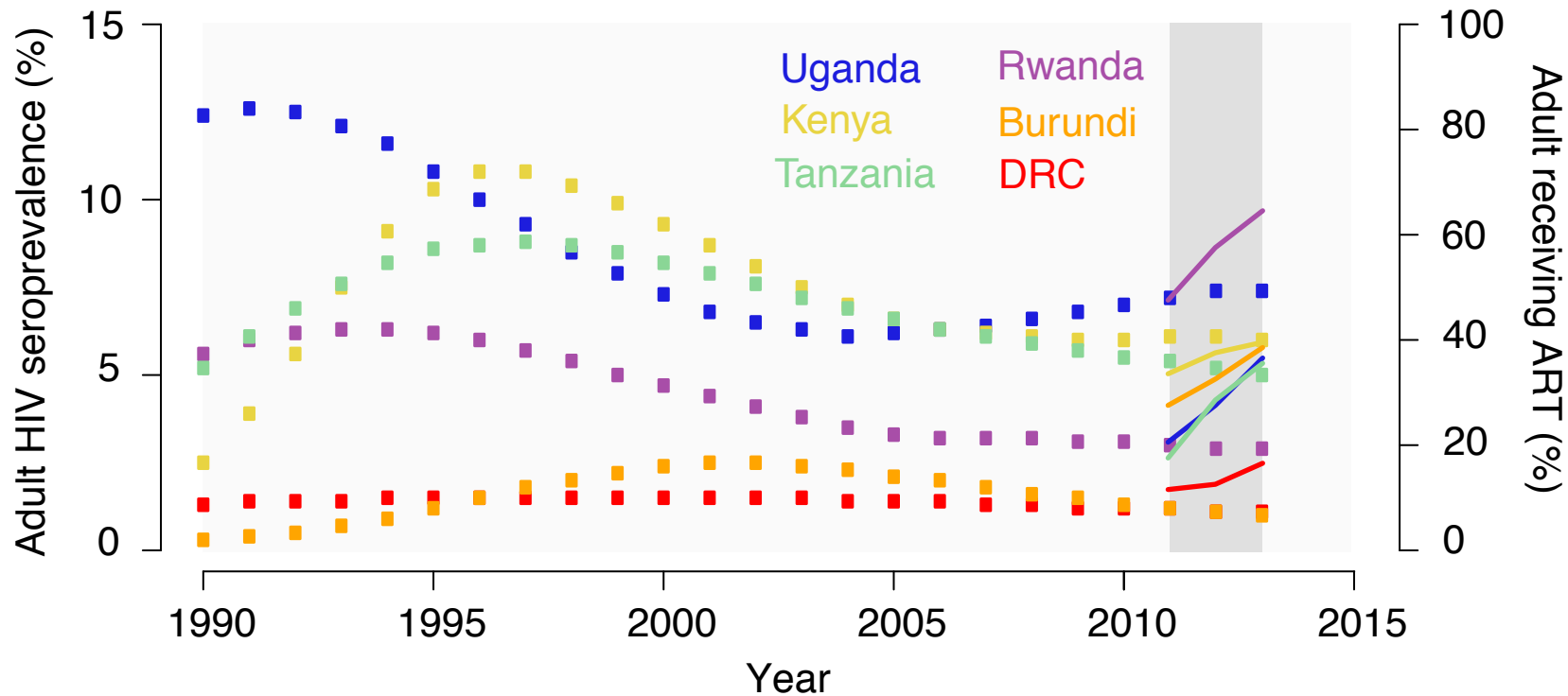

Supplement: S5 Fig — (PDF) [file ppat.1007976.s017.pdf]

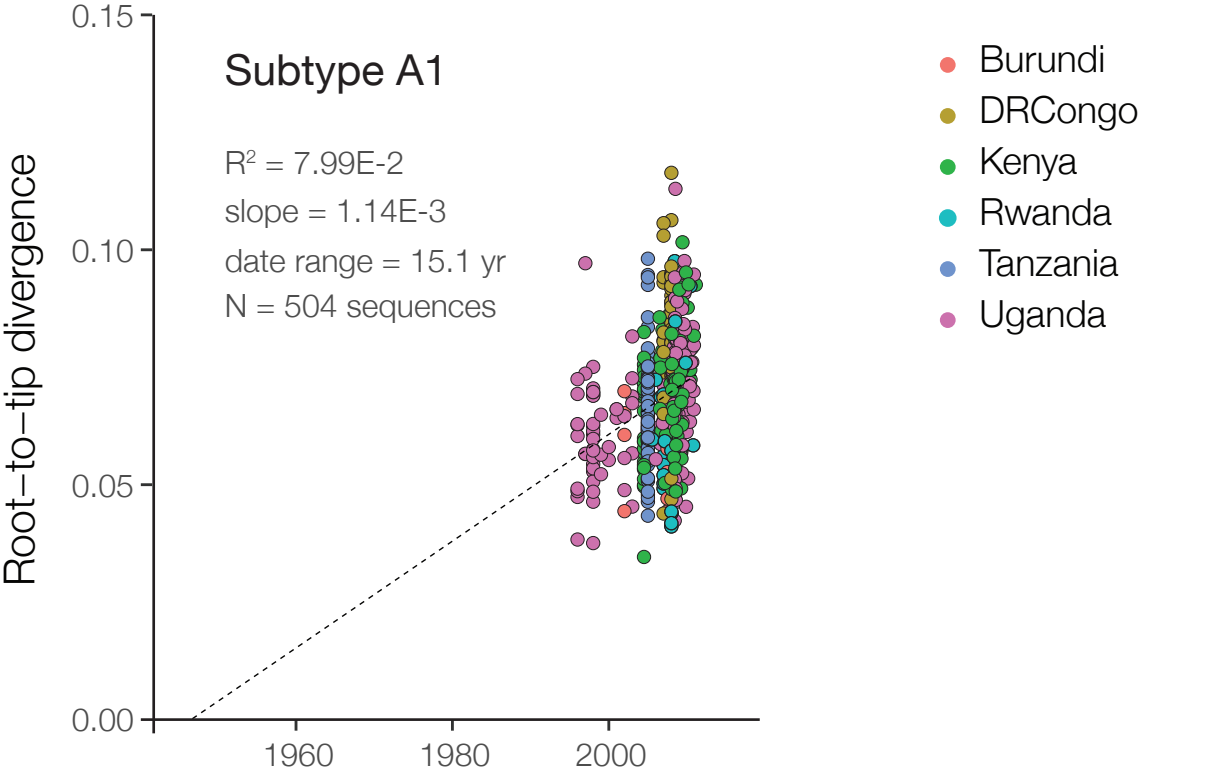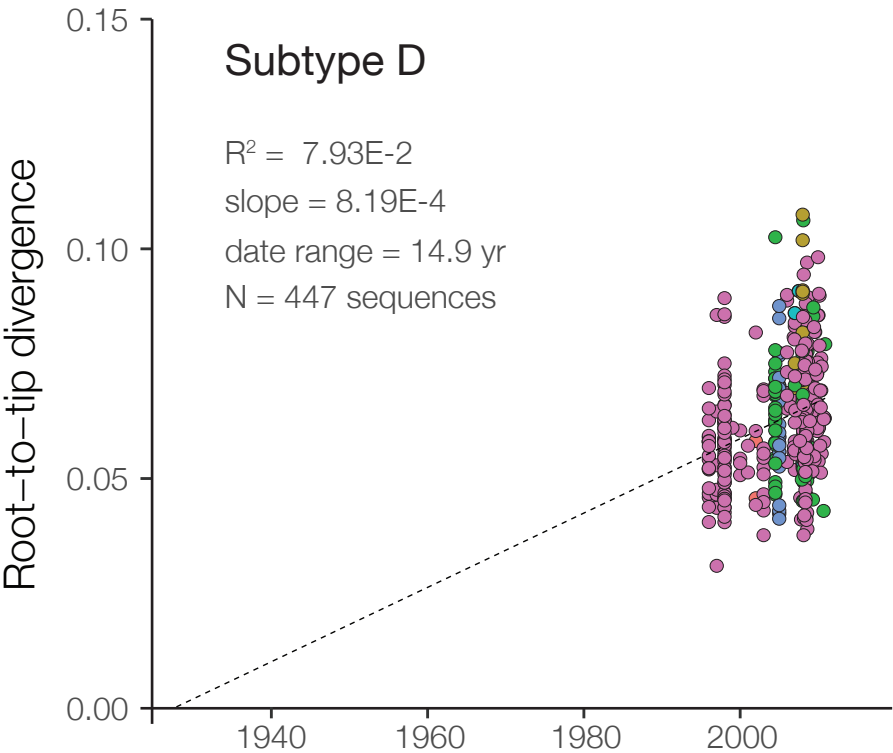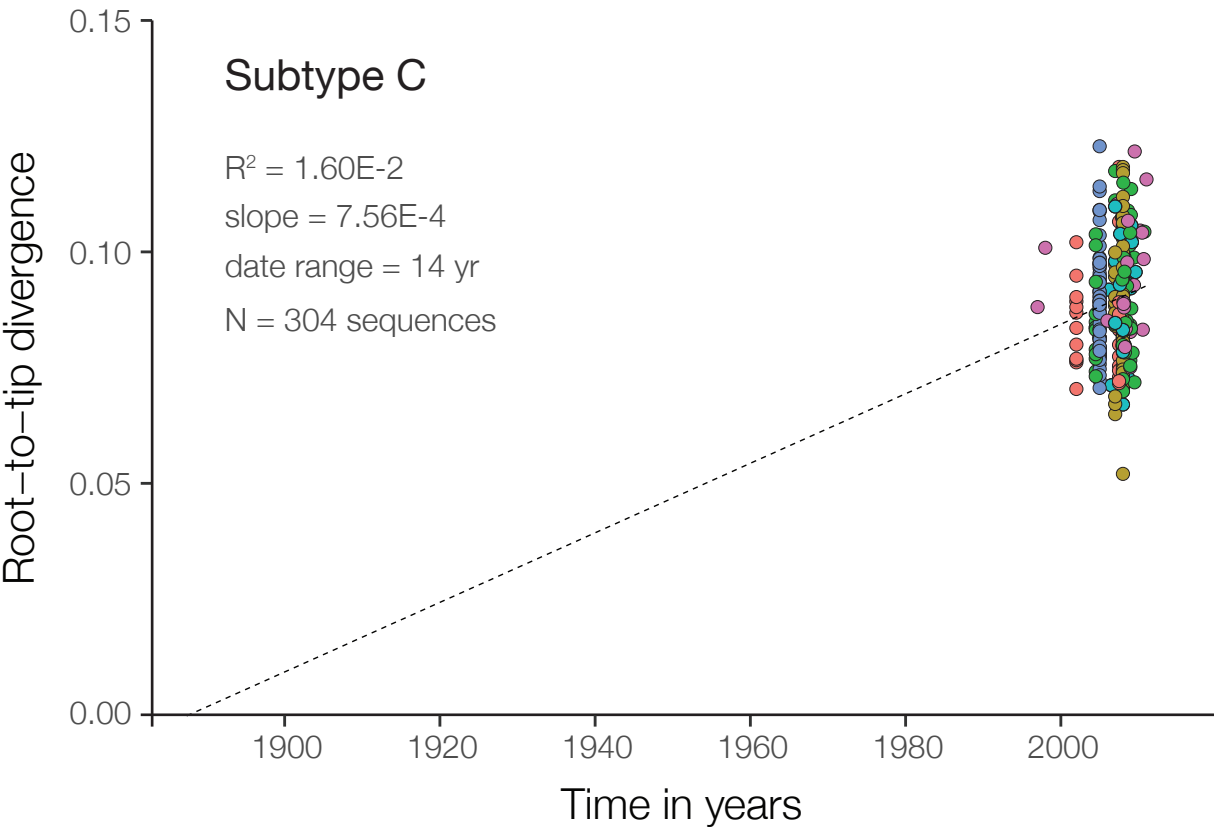

Supplement: S6 Fig — Correlation and determination coefficient (R2) were estimated with TempEst. (PDF) [file ppat.1007976.s018.pdf]

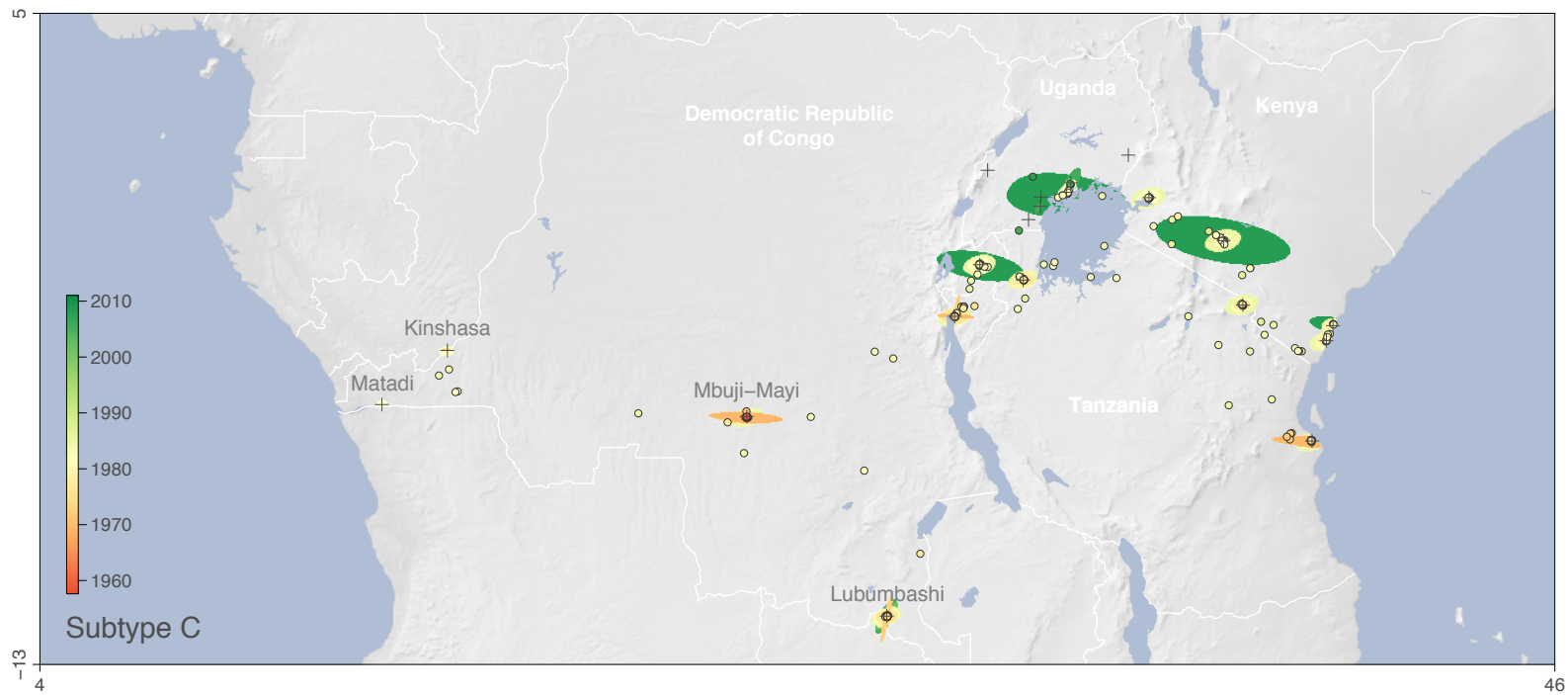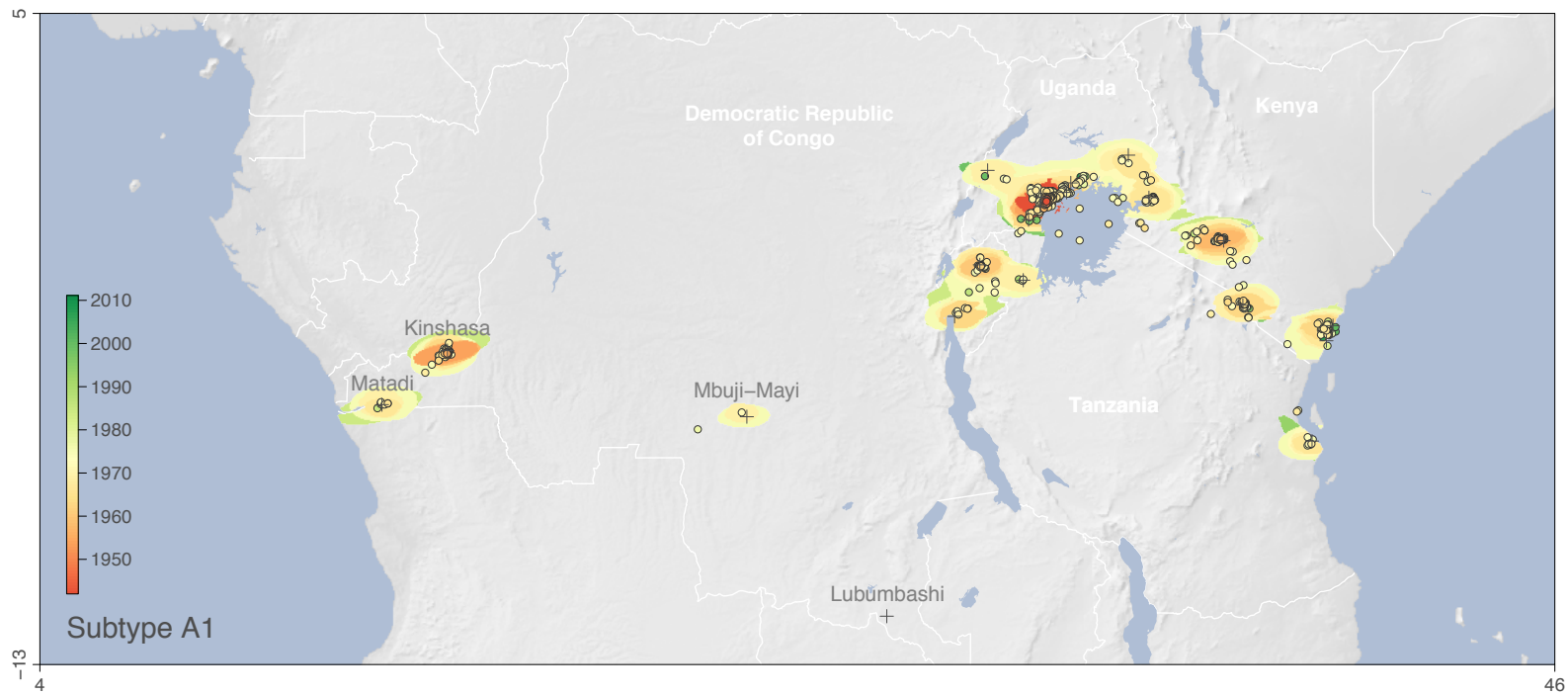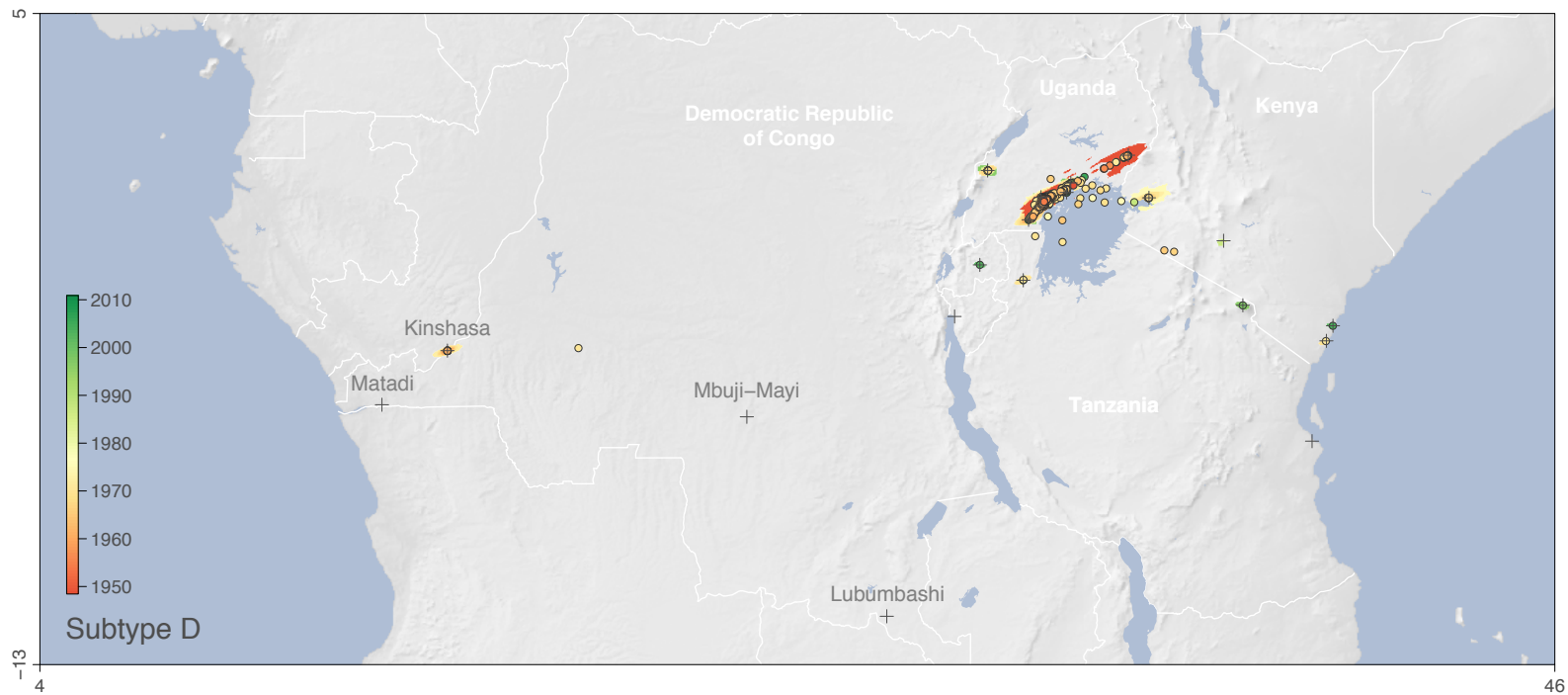

Supplement: S7 Fig — Internal nodes of maximum clade credibility and 95% HPD regions based on 1,000 trees subsampled from the posterior distribution of each continuous phylogeographic analysis. MCC tree internal nodes are coloured according to their time of occurrence, and 95% HPD regions were computed for successive time layers and then superimposed using the same colour scale reflecting time. Crosses indicate the position of the sampling locations. (PDF) [file ppat.1007976.s019.pdf]

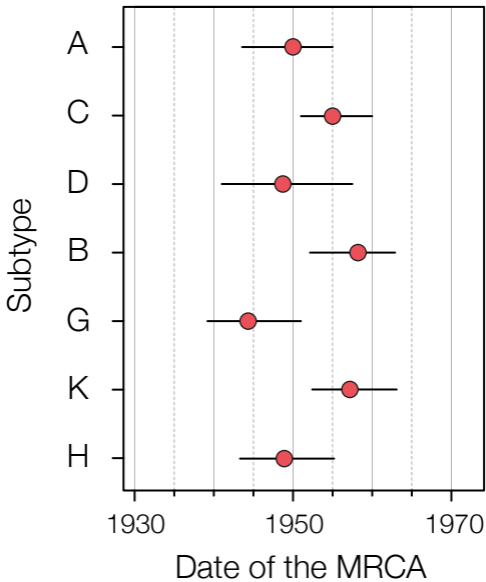

Supplement: S8 Fig — These values are used to define normal priors for TMRCA parameters estimated in BEAST analyses (see the text for further details). (PDF) [file ppat.1007976.s020.pdf]
